# Downregulation of cyclic adenosine monophosphate levels in leukocytes of hibernating captive black bears is similar to reported cyclic adenosine monophosphate findings in major depressive disorder

**DOI:** 10.3389/fpsyt.2023.1123279

**Published:** 2023-03-16

**Authors:** John A. Tsiouris, Michael Flory

**Affiliations:** ^1^George A. Jervis Clinic, New York State Institute for Basic Research in Developmental Disabilities, Staten Island, NY, United States; ^2^Department of Psychiatry, State University of New York Downstate Medical Center, Brooklyn, NY, United States; ^3^Research Design and Analysis Service, New York State Institute for Basic Research in Developmental Disabilities, Staten Island, NY, United States

**Keywords:** cAMP downregulation, leukocytes, hypometabolism, mammalian hibernation, black bears, major depressive disorder

## Abstract

**Introduction:**

Cyclic adenosine monophosphate (cAMP) levels in the lymphoblasts and leukocytes of patients with major depressive disorder (MDD) have been reported to be downregulated compared to in controls. cAMP is a derivative of adenosine triphosphate (ATP), and low ATP turnover has been reported in the state of hypometabolism associated with human MDD and with mammalian hibernation due to suppression of mitochondrial metabolism. Similarities have been noted between many state-dependent neurobiological changes associated with MDD in humans and with mammalian hibernation.

**Methods:**

To compare cAMP levels between human MDD and mammalian hibernation and to investigate whether cAMP downregulation is another state-dependent neurobiological finding, we measured cAMP concentrations in lysed leukocytes, plasma, and serum in serial blood specimens from nine female captive black bears (*Ursus americanus*; CBBs), and cortisol levels in serum from 10 CBBs.

**Results:**

Cortisol levels were significantly higher during hibernation in CBBs, confirming previous findings in hibernating black bears and similar to findings in humans with MDD. cAMP levels were significantly lower during hibernation versus active states (pre-hibernation and exit from hibernation) and were similar to the cAMP downregulation reported in MDD patients versus euthymic patients or controls. cAMP level changes during the different states (hibernation, pre-hibernation, active) confirm their state-dependent status.

**Discussion:**

These findings are similar to the neurobiological findings associated with the hypometabolism (metabolic depression) observed during mammalian hibernation and reported during MDD. A sudden increase in cAMP levels was observed before entrance into pre-hibernation and during exit from hibernation. Further investigation is suggested into the possible role of elevated cAMP levels in initiation of the chain reaction of changes in gene expression, proteins, and enzymes leading to the suppression of mitochondrial metabolism and to low ATP turnover. This process leads to hypometabolism, the old adaptive mechanism that is used by organisms for energy preservation and is associated with both mammalian hibernation and human MDD.

## Introduction

The cyclic adenosine monophosphate (cAMP) signaling pathway is a second messenger, and because its regulation at the intracellular level is critical for the transduction of cellular response, the role of cAMP generation in bipolar disorder and especially major depressive disorder (MDD) has been studied extensively [see review, (1)]. Most of these studies, using different materials, specimens, and methods, reported a downregulation in the production of cAMP in patients with MDD versus normal controls and patients in a euthymic or a manic state (1).

Mean levels of cAMP in the plasma of patients with bipolar disorder were not different between the different phases of their illness or compared to controls (2). Beta adrenoreceptor–mediated cAMP formation, using histamine, prostaglandin E1, or isoproterenol, was not different in the lymphoblasts of depressed patients versus controls (3), and no difference was found either in the affinity or the density of beta receptors on lymphoblasts between patients with bipolar disorder and controls (3, 4). Studies of histamine-stimulated and beta adrenoreceptor–mediated cAMP formation in leukocytes from patients with MDD and controls reported lower cAMP levels (i.e., downregulation) in patients with MDD versus controls (5–7). When cAMP formation was measured in isoproterenol-stimulated lymphocytes and not in leukocytes, lower cAMP levels were found only in patients with MDD and psychomotor agitation, not in patients with psychomotor retardation (8, 9). Reduction in beta-adrenoreceptor–linked cAMP-dependent protein kinase (PKA) was reported in patients with MDD and melancholic features versus controls (10), suggesting low levels of cAMP.

The heterogeneity of the subjects and the differences in disease severity, in the specimens in which cAMP was measured (plasma, stimulated leukocytes/lymphocytes, and platelets), and in the methods used for determination of cAMP levels may have contributed to the different, and in certain studies, opposite, findings (1).

The theory of cAMP dysfunction as a cause of MDD and of depression in general was proposed (11) on the basis of findings of cAMP-signaling downregulation in MDD and its upregulation with chronic administration of antidepressants in one postmortem study in humans (12) and in studies in rodents (13). This theory was further supported by findings of decreased cAMP signaling in the brains of unmedicated depressed patients and increased cAMP signaling after treatment with a selective serotonin reuptake inhibitor using _11_C-(R)-rolipram PET scan to image phosphodiesterase 4 (PDE4) (14). Neither of these studies nor any cAMP studies investigated whether the reported changes are state-dependent or not.

The mitochondrial dysfunction theory of MDD was proposed to explain the downregulation of cAMP and the hypometabolism observed during MDD (15, 16). However, recent studies have linked suppression of mitochondrial function, including low adenosine triphosphate (ATP) turnover, but not mitochondrial dysfunction, to MDD and have suggested the need for further investigation of mitochondrial changes during MDD (17–20).

A theory explaining the origin of the hypometabolism observed during MDD was proposed by the first author in 2005 (21), arguing that a *form* of metabolic depression, a primitive adaptive process for energy preservation that is homologous to the one responsible for mammalian hibernation—and not mitochondrial dysfunction—is the process underlying the observed hypometabolism, state-dependent neurobiological changes (22, 23), and vegetative symptoms of MDD in humans. Supporting evidence for this metabolic depression theory of MDD and for its link to the process of mammalian hibernation (21) is the finding that suppression of mitochondrial metabolism is the process responsible for mammalian hibernation (24, 25), including low ATP turnover (26, 27), as well as the similarities to the findings from mitochondrial studies in MDD (17–20).

The following state-dependent neurobiological changes that have been documented as being similar between MDD in humans and mammalian hibernation offer further support for the metabolic depression theory of MDD (21): reversible subclinical hypothyroidism (28, 29), increased serum cortisol concentration (30–32), acute phase protein response (33–38), low respiratory quotient (39–42), oxidative stress response (43–45), and decreased neurotransmitter levels (39, 46, 47). Black bears (*Ursus americanus*) were suggested and used as an animal model for MDD to study the process of hypometabolism during hibernation (21) because many of the similar neurobiological changes between MDD and mammalian hibernation had been observed in studies of bears, especially black bears, during hibernation (29, 32, 36, 37, 40, 45). The full rationale for using this model has been explained previously [(21); see pp. 830–831]. Black bears in the wild withdraw into dens in October, and by November 1st, most are in the state of hibernation proper without becoming hypothermic (32, 40, 41). Hypothermia, which is common in all other hibernating mammals, suppresses gene expression and the translation and transcriptional machinery of many proteins (48).

Mammalian hibernation is a state of metabolic stress, which leads to the dephosphorylation of ATP through the activation of adenylyl cyclase and the formation of cAMP, which is converted to adenosine 5’ nucleotidase (5’ AMP) (1, 49). According to these findings (49), higher cAMP concentration would be expected in the lysed leukocytes of black bears before entrance into hibernation, the basic state of hypometabolism, when the process shifts from normal to the state of metabolic depression, and low cAMP levels are expected during hibernation proper. To compare our expected findings of cAMP level changes during mammalian hibernation with the reported findings during MDD in humans, to investigate the state-dependent status of cAMP levels, and to obtain additional supporting evidence for the metabolic depression theory of MDD (21), we measured the baseline changes of cAMP levels in serial blood samples obtained every 15 days during active, pre-hibernation, and hibernation states from nine female captive black bears (CBBs) kept in captivity from October to May 15. Serum cortisol levels also were measured from the serial samples obtained from these same CBBs plus one additional CBB. We report our findings here.

## Materials and methods

### Materials

The cyclic AMP (direct) colorimetric ELISA kit and the cortisol ELISA kit were obtained from Assay Designs Inc. (Ann Arbor, MI, United States). All other chemicals were of reagent grade.

### Subjects

Serial blood samples were obtained every 15 days from 11 female CBBs 3 to 21 years of age (mean age 11, SD 6.67), six of which were pregnant. These CBBs were kept in captivity from October to April at the Center for Ursid Research at Virginia Polytechnic Institute and State University (VPI) in Blacksburg, VA, United States. One of these CBBs was captive from October 2002 to May 15, 2003; seven, from October 2003 to May 15, 2004; and three, from October 2004 to May 15, 2005. Ages of the CBBs from which specimens were obtained for analysis were 3–8 years (*n* = 3 for cAMP levels, and *n* = 4 for cortisol levels), 9–15 years (*n* = 4), and older than 15 years (*n* = 2). Although specimens were obtained from 11 CBBs from October 2002 to May 2005, only nine were included in the analyses for cAMP levels, and 10 for serum cortisol levels. One CBB, 2 years of age, was excluded from the cortisol and cAMP level analyses because it never fell into hibernation, and a second one was excluded from cAMP analyses because only one cAMP specimen was obtained. During the active state after captivity, in October, only a few cAMP specimens were received and thus were excluded from analysis. The serum cortisol specimens obtained in October also were excluded from analysis, because of the sudden increase in levels resulting from the stress of captivity.

### Trapping and immobilization of animals

All of the CBBs were captured in the George Washington National Forest and Jefferson National Forest in western Virginia. They were trapped in either Aldrich leg-hold snares or culvert traps, and all were anesthetized with a 2:1 mixture of ketamine hydrochloride (Ketaset^®^, Fort Dodge Animal Health, Fort Dodge, IA, United States) and xylazine hydrochloride (Rompum^®^, Bayer Corporation, Shawnee Mission, KS, United States) (concentration 300 mg/mL) at a dose rate of 1 mL/45 kg body mass. Telazol^®^ was not used. No CBBs were in a trap for more than 20 h, and most were in traps for less than 12 h. All CBBs were healthy when the blood samples were drawn. Protocols for handling wild black bears and CBBs were approved by the VPI Animal Care Committee and the Institutional Animal Care and Use Committee at the New York State Institute for Basic Research in Developmental Disabilities (IBR). For further details, see Tsiouris et al. (37) and Donahue et al. (50).

Captive black bears entered hibernation proper between late December and January 1—not by November 1, as in most black bears in the wild (32, 41)—because of food availability and the higher temperature of the quarters in which they were kept captive. Each CBB was fed a daily ration of 2,000 g of dry high-protein (23%) dog food until November 30. Rations were cut to 1,000 g on December 1, to 500 g on December 10, and to 250 g on December 20. Feeding was terminated on December 30 and not resumed until April 1. Pregnant CBBs gave birth to their cubs during the last week of January without exiting the hibernation state. CBBs exited the hibernation state and became active in late March through April 1st similarly to black bears in the wild.

### States during captivity

Active State: After captivity; October 1–October 31.

Pre-hibernation State: November 1–December 18 (early December).

Proper Hibernation State: December 19 (late December)–March 18 (early March).

Exit from hibernation/Active State: March 19 (late March)–May 15.

### Black bear blood and sera

The number of blood specimens obtained from the nine CBBs for cAMP levels was 58, and of serum specimens obtained from 10 CBBs for cortisol levels was 137. Sera were prepared on-site and stored frozen at VPI until shipped. Whole-blood samples were collected in heparinized tubes and immediately shipped overnight to IBR on ice packs. Because only one specimen was obtained from each of four CBBs at the end of April after exit from hibernation, they were not included in the analysis.

### Harvesting of black bear leukocytes

Whole blood was centrifuged for 10 min @ 2,000 × g (4^°^C). The buffy coat was collected and washed once in tris-buffered saline (TBS). The cells were collected by centrifugation, and the pellets were suspended in TBS, aliquoted, and stored at −80^°^C until needed.

### Assay of protein concentration

Protein concentrations were determined by the method of Lowry et al. (51) by using bovine serum albumin as standard.

### cAMP levels in black bear leukocytes

#### Preparation of leukocytes

Blood was drawn from female CBBs in heparinized (green-top) tubes and shipped by overnight express from VPI to IBR. The tubes were centrifuged at 800 × g for 15 min at room temperature (RT). The plasma and buffy-coat were removed to clean tubes and were centrifuged at 2,000 × g for 15 min at RT. The plasma was removed, and the cells were washed with an isotonic tris-buffered saline (TBS; pH 8.0) and re-centrifuged. The washing was repeated once. The cells were re-suspended in TBS, quick-frozen on dry ice, and stored at −80^°^C.

#### Assay of adenosine 3’, 5’–cAMP

The concentration of cAMP in leukocytes was determined with direct cAMP colorimetric ELISA kit (Assay Designs, Inc., Ann Arbor, MI, United States), following the manufacturer’s protocol. This kit is designed for use with cell culture and tissue and includes 0.1 M HCl, which is used to lyse the cells, stop endogenous PDE activity, and stabilize the released cAMP. The 0.1% Hcl-treated samples are then analyzed directly in a microtiter plate without extraction, drying, and reconstitution. Because the sample-to-sample yield of leukocytes was variable, aliquots of the cells were removed to measure the total protein concentration, after which the cAMP values were normalized to 100 μg of total protein.

### Cortisol levels in black bear sera

The levels of cortisol in black bear sera were determined by using the cortisol ELISA kit (Assay Designs, Inc.) following the manufacturer’s protocol.

## Results

Assays of cAMP levels were performed on 137 serum samples, 58 plasma samples, and 58 samples of lysed leukocytes. Levels of cAMP from plasma samples and from lysed leukocytes were positively correlated (r = 0.49, *p* = 0.0001, *N* = 57), although samples from serum were negatively correlated with measurements of both plasma (r = −0.50, *p* = 0.0002, *N* = 50) and lysed leukocytes (r = −0.33, *p* = 0.02, *N* = 51). However, these correlations are in large part autocorrelations among repeated measures; correlations adjusted for autocorrelation are much lower (lysed leukocytes and plasma, rho = 0.07, *p* = 0.62; lysed leukocytes and serum, rho = −0.01, *p* = 0.94; plasma and serum, rho = −0.004, *p* = 0.9815) (52, 53). Measurements of cAMP levels in lysed leukocytes were used for analyses for both robustness of measurements and comparison with published research.

Before the analyses were performed, distributions of cAMP and cortisol measurements were tested for normality using the Shapiro-Wilk W test. Measurements of both factors were found to be severely skewed to the right. A square-root transformation was applied to both variables, making them acceptably normal. Comparisons of groups were performed using generalized estimating equations (GEEs) (54, 55) to avoid the distortion of variance estimates arising from autocorrelation of each bear’s multiple measurements. Robust estimates of variance were employed in all analyses. Analyses were performed using Stata version 16.1 (56).

Tables 1, 2 provide the number of measurements, their means, and the standard deviations for cortisol and cAMP levels by state of hibernation, overall, and divided by pregnancy status and age group.

**TABLE 1:** Cortisol levels (ng/ml) by pregnancy status and age.

		State of hibernation														
		**Active** **(Oct 1–Oct 31)**			**Pre-hibernation** **(Nov 1–Dec 18)**			**Hibernation** **(Dec 19–Mar 18)**			**Exit** **(Mar 19–May 15)**			**Total**		
		***N***	**Mean**	**SD**	***N***	**Mean**	**SD**	***N***	**Mean**	**SD**	***N***	**Mean**	**SD**	***N***	**Mean**	**SD**
Total		4	5.78	5.31	30	2.59	3.40	65	4.28	4.71	38	3.71	4.75	137	3.80	4.50
Pregnant?	No	1	8.82	–	8	0.90	0.65	19	1.75	1.96	7	0.55	0.43	35	1.51	2.01
	Yes	3	4.77	6.01	22	3.20	3.79	46	5.32	5.12	31	4.43	4.99	102	4.58	4.84
Age group (years)	3–8	2	6.46	7.42	14	1.97	3.07	25	4.02	5.78	18	2.95	4.38	59	3.29	4.85
	9–15	2	5.10	5.26	13	2.61	3.84	28	4.11	3.76	13	3.79	4.46	56	3.72	3.93
	>15	–	–	–	3	5.36	1.99	12	5.20	4.50	7	5.54	6.28	22	5.33	4.72

*N*, number of measurements; SD, standard deviation.

**TABLE 2:** Cyclic adenosine monophosphate (cAMP) levels (pmol/ml) by pregnancy status and age.

		State of hibernation														
		**Active** **(Oct 1–Oct 31)**			**Pre-hibernation** **(Nov 1–Dec 18)**			**Hibernation** **(Dec 19–Mar 18)**			**Exit** **(Mar 19–May 15)**			**Total**		
		***N***	**Mean**	**SD**	***N***	**Mean**	**SD**	***N***	**Mean**	**SD**	***N***	**Mean**	**SD**	***N***	**Mean**	**SD**
Total		2	24.48	3.13	10	50.62	27.74	30	17.44	13.97	16	30.37	29.23	58	26.97	24.40
Pregnant?	No	1	26.70	**–**	6	37.29	13.49	11	20.17	8.27	6	43.94	33.61	24	30.66	20.63
	Yes	1	22.27	**–**	4	70.63	33.39	19	15.86	16.41	10	22.23	24.50	34	24.37	26.73
Age group (years)	3**–**8	**–**	**–**	**–**	2	28.38	13.13	11	15.60	11.85	7	21.74	15.34	20	19.03	13.26
	9**–**15	2	24.48	3.13	8	56.18	28.06	14	19.11	16.94	7	44.40	37.95	31	34.73	29.36
	>15	**–**	**–**	**–**	**–**	**–**	**–**	5	16.83	10.57	2	11.50	12.62	7	15.31	10.38

*N*, number of measurements; SD, standard deviation.

**FIGURE 1:**
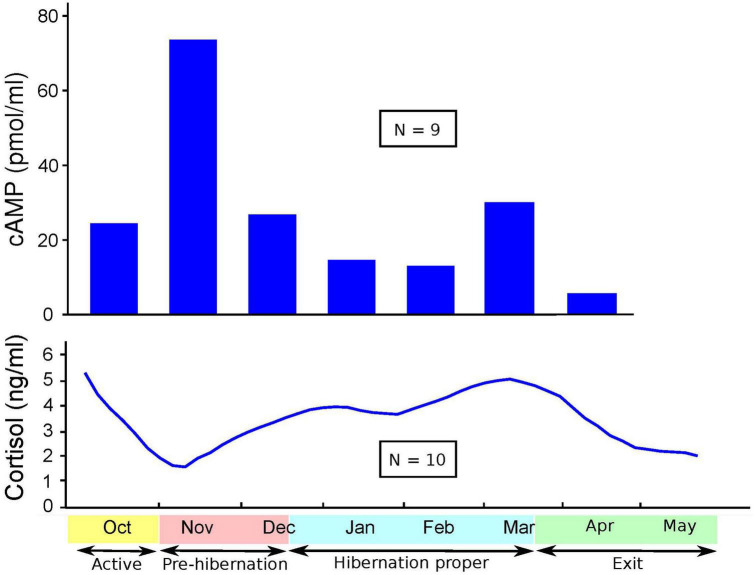
Levels of cyclic adenosine monophosphate (cAMP) and cortisol over time.

**FIGURE 2:**
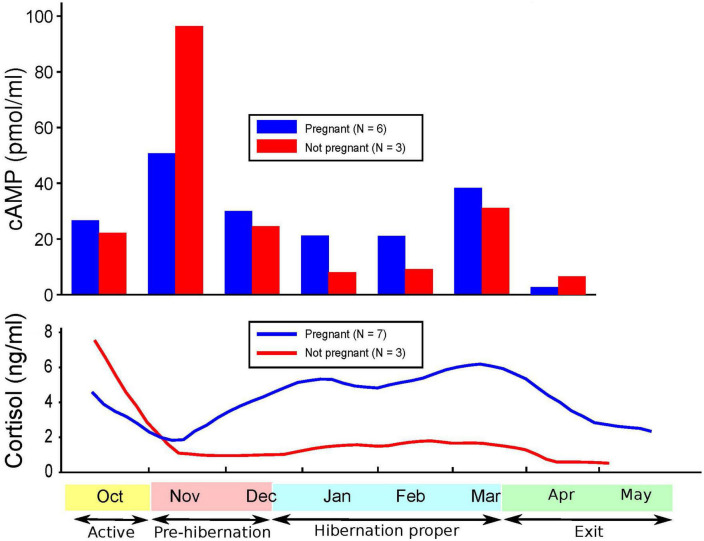
Levels of cyclic adenosine monophosphate (cAMP) and cortisol over time and pregnancy status.

**FIGURE 3:**
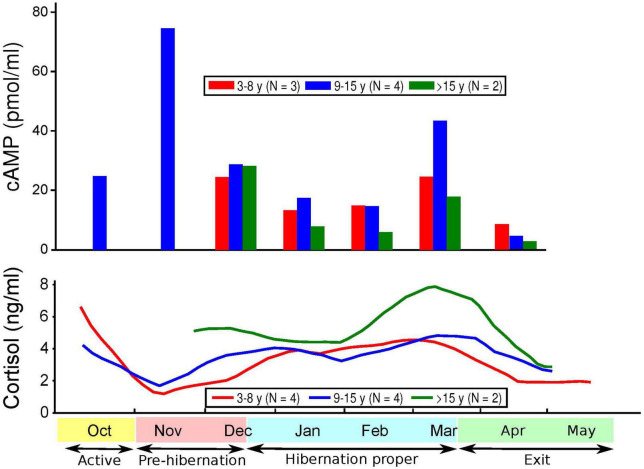
Levels of cyclic adenosine monophosphate (cAMP) and cortisol over time and age of subjects.

Figures 1–3 show cortisol and cAMP levels throughout captivity, overall, and divided by pregnancy status and age group.

Cortisol levels were found to be very high in the sera of the CBBs after their captivity. The levels of cortisol decreased in the weeks after the start of captivity during the month of November, increased during hibernation, and decreased again after the exit from hibernation into the active state. The age and pregnancy status of the CBBs influenced the levels of cortisol, but not the direction of level changes, in the sera during the active and hibernation states. Cortisol levels were significantly higher during hibernation (mean 4.28, SD 4.71, *N* = 65) than during pre-hibernation and the exit state immediately following hibernation (mean 3.22, SD 4.22, *N* = 68, Wald chi2(1) = 9.88, *p* = 0.0017).

Cyclic adenosine monophosphate levels in the lysed leukocytes of CBBs were higher in the active state after captivity and before the entrance into pre-hibernation (November and early December) than during the hibernation state (late December–early March). Levels increased again moderately at the end of hibernation (late March) during exit from hibernation (active state). The age and pregnancy status of the CBBs influenced the levels of cAMP, but not the direction of level changes, in the lysed leukocytes during the different active and hibernation states. Levels of cAMP were significantly lower during hibernation (mean 17.44, SD 13.97, *N* = 30) than during pre-hibernation and the exit state immediately following hibernation (mean 38.16, SD 29.84, *N* = 26, Wald chi2(1) = 4.24, *p* = 0.0395).

Thus, the changes in the levels of cAMP in lysed leukocytes and of cortisol in serum were in opposite directions with entry into pre-hibernation: cAMP levels increased, and cortisol levels decreased. Both levels increased in late March during the exit from hibernation.

## Discussion

Our findings of significantly increased cortisol levels in the serum of hibernating CBBs confirm similar previous findings (32, 57) and are similar to the increased cortisol levels reported in the severe type of MDD in humans (30, 31).

The high levels of cortisol measured in the serum of the CBBs immediately after their captivity are due to the glucocorticoid stress responses to the capture of wild, free-living mammals (58) and have similarities to the stress response in humans (59).

Our findings of significantly lower cAMP levels in CBBs during hibernation versus their active state are similar to the findings of lower cAMP levels in humans with MDD versus in controls (5–7, 10) and in humans with MDD with psychomotor agitation (8). The lack of differences reported in the cAMP levels in the plasma of patients in different states (depressed, manic, euthymic) and matched controls (2) could be explained by our findings of a very weak correlation between the cAMP levels in leukocytes and plasma in CBBs. These findings suggest that plasma is not a good medium for documenting changes in cAMP levels in different states. cAMP levels in the serum of CBBs were negatively correlated with both plasma and leukocyte cAMP levels. Also, our findings were opposite to the lack of differences observed in cAMP production between patients diagnosed with schizophrenia or MDD versus drug-free normal controls (60). The increase in cAMP levels during the entrance into pre-hibernation, especially in pregnant and 5–15 years-old CBBs, and the decrease in cAMP levels in the following weeks are a new finding, which must be replicated in male and female black bears in the wild and in captivity, and in humans when entering into severe MDD, especially with melancholic/catatonic features.

The age and pregnancy status of CBBs influenced the degree of changes in both cortisol and cAMP levels but not the direction of the changes. Similar variability in the findings has been reported in cAMP levels in human MDD (1).

Metabolic stress triggers the dephosphorylation of ATP and the formation of cAMP (49). Whether the increase in cAMP levels in CBBs in the month of November is associated with low levels of ATP due to dephosphorylation and formation of cAMP in mammalian hibernation (27, 58, 59) and possibly in MDD (17–20) must be investigated.

Low cAMP levels, which decrease activation of PKA, and lower ATP turnover are both associated with reduced energy production, which leads to hypometabolism (metabolic depression), the process underlying mammalian hibernation (24, 26, 27, 61, 62). There are no published studies measuring cAMP level changes during active or hibernation states in bears or other large mammals that hibernate without becoming hypothermic (40) against which our findings can be compared. Research will be required to confirm our findings and their similarities with the findings in MDD.

Research also will be required to determine whether the drop in cAMP level after its increase with the initiation of pre-hibernation in CBBs and with the possible initiation of hibernation in black bears in the wild is associated with the increased production of 5’ AMP through the enzymatic hydrolysis of cAMP carried out by PDE4. PDE4 cleaves the 3;5’ PDE bond in cAMP, thus inhibiting the phosphorylating activity of PKA (49).

5’ AMP levels have been found to be elevated in mice during dark/dark periods, and injection of 5’ AMP in light/dark periods in non-hibernating mice produced torpor-like changes (63). 5’ AMP has been reported to be one of the triggers (circadian signals) for the initiation of the hypometabolic state and the molecular process involved in 5’ AMP-induced hypometabolism, but this process occurs at the metabolic interconversion level rather than at the gene or protein expression level, as it has been observed that widespread suppression of energy-generating metabolic pathways occurs during this process (64).

Hypometabolism is the result of reduced energy production (19), which occurs secondary to decreased ATP turnover respiration in depressed patients versus controls (18), and to decreased brain levels of ATP and differentially expressed proteins in depressed patients, which are mainly related to deregulation (downregulation) or energy metabolism pathways (17).

The major characteristics of MDD in humans, as per the DSM-5 (65), are lack of energy, fatigue, lack of interest, lack of motivation, avoidance of interaction, weight loss, insomnia, and psychomotor retardation, all of which are secondary to hypometabolism and are associated with low ATP turnover (18, 19). This condition, according to Engel and Schmale (66), was called “conservation withdrawal.” It appears that if untreated, some patients with MDD develop melancholic or catatonic features (the prototype of metabolic depression in MDD) or a constant state of agitation possibly dependent on their temperament (genetics), personality features, and environmental stressors. The symptoms of diminished ability to think are secondary to lack of energy, and the depressed mood, feelings of worthlessness, hopelessness, or excessive guilt appear to be secondary to the negative reevaluation of self, rumination of past actions perceived as negative due to the lack of energy and strength and to weakness, for which patients blame themselves, as often, no medical explanation for their condition is possible. The symptoms of suicidal phenomena (suicidal ideation, suicide threats/attempts, and suicide death) have been considered to be plans and actions of the person asking for help, trying to escape from or terminating the unbearable condition created by the core symptoms associated with hypometabolism. The findings of cAMP level changes in response to treatment of humans with MDD with antidepressants (12–14) and of changes in the levels of cAMP in different states (pre-hibernation, hibernation, and active) in CBBs suggest the state-dependent quality of cAMP, as was reported previously (5), and are similar to the other neurobiological changes associated with MDD in humans and with hibernation in mammals investigated to date (21). The decreased cAMP levels in CBBs during hibernation and in patients with MDD suggest similarities in the cAMP levels in the two conditions and can be added to the observations of many neurobiological changes that are similar in the two conditions (21). These findings do not support the theory of cAMP dysfunction in MDD in humans that has been proposed by Fujita et al. (11, 14).

Our study has the following limitations: the small total number of CBBs from which specimens were obtained, although the adequate number of serial specimens obtained compensated for this; the sample comprising entirely female CBBs, two-thirds of which were pregnant; the captivity status; the few specimens obtained for determination of cAMP levels during the active state after the CBBs’ exit from hibernation; and that cAMP levels were analyzed for lysed leukocytes during hibernation and the states before it and after exit from it in CBBs, which was different from the methods used to determine cAMP levels in lymphoblasts, lymphocytes, and leukocytes in only one specimen obtained from patients with MDD versus euthymic patients or controls. However, the longitudinal nature of the data collected and the analysis and comparison of both cAMP and cortisol levels in this study are unusual if not unique, offering insight into both the entry into, and the emergence from, a state resembling, in some ways, MDD in humans. Also, this is the first study to document cAMP level changes in the leukocytes of black bears during active and hibernation states, revealing their similarities with the cAMP level changes reported in patients with MDD versus controls, and confirming the state-dependent changes of cAMP.

In conclusion, this study is the first to document changes in cAMP levels during mammalian hibernation and has established that the downregulation of cAMP levels is similar during hibernation of female CBBs and in patients with MDD; that there is a significant increase in cAMP levels with the entrance to the pre-hibernation state during captivity in the month of November, which corresponds to the month of entrance into hibernation proper of black bears in the wild, whereas the levels of serum cortisol are low (opposite direction); and that there is a mild increase in cAMP levels during the exit from hibernation (late March), whereas the serum cortisol levels are high, as they have been during hibernation proper. Finally, this study strongly suggests that the changes in cAMP levels in CBBs are state-dependent, as are most of the similar neurobiological findings associated with MDD in humans and mammalian hibernation. These findings dispute the theory of cAMP dysfunction as a cause of MDD, as was recently suggested (11, 14).

In-depth studies of the biological process of mammalian hibernation are under way (24, 25, 64, 67). Research on the cause of hypometabolism in MDD in humans has recently questioned the mitochondrial dysfunction theory of MDD and has suggested instead that suppression of mitochondrial function, which leads to the low ATP turnover observed in MDD, is the cause of hypometabolism in MDD (17–20). The findings of downregulation of cAMP levels during hibernation in female CBBs must be replicated in lysed leukocytes and other cells obtained through serial specimens from black bears (male and female) and from other hibernating mammals in the wild and in captivity, and from patients with recurrent MDD in different states of severity and in euthymic states. Confirmation of our findings, together with the previous findings of low ATP turnover in MDD (17–20), would offer more supportive evidence of the theory that a *form* of metabolic depression that is homologous to the one responsible for mammalian hibernation is the underlying adaptive process responsible for MDD in humans (21).

In spite of vigorous research, a full understanding of the etiology and neurobiology of MDD has been elusive to date (68), and no explanatory theory exists for most of the abnormal findings that have been proven to date to be state-dependent. The similarities in the findings of research regarding suppression of mitochondrial metabolism and neurobiological changes between human MDD and mammalian hibernation call for a paradigm shift in MDD research.

This new paradigm must seriously consider that the core symptoms of MDD are secondary to hypometabolism, a *form* of metabolic depression (21) homologous to one that is responsible for mammalian hibernation and also for torpor and estivation in many organisms (27, 64, 69, 70). It is hoped that this paradigm will inspire new basic research into the process of entering into and exiting from the state of hypometabolism in both MDD and mammalian hibernation and an extensive collaboration between researchers involved in the study of hypometabolism in these conditions. Understanding and viewing MDD as an old adaptive process that is activated in about 20% of humans under different adverse conditions (71) could open many new pathways for research into the etiology and neurobiology of MDD. We propose again [as previously (21)] that the process responsible for the documented hypometabolism in mammalian hibernation—suppression of mitochondrial function—is partially or fully activated in 20% of humans in their lifetime (71), leading to MDD, especially in humans exposed to environmental stressors (72, 73) and predisposed to MDD by behavior inhibition by temperament (74), anxiety disorders (75), and early life traumatic experience (76).

If future research fully confirms the theory that a form of hypometabolism that is homologous to the one responsible for mammalian hibernation is the underlying old adaptive process responsible for human MDD, the stigma associated with MDD could be reduced, and our diagnostic and treatment strategies for MDD could be improved.

## Data availability statement

The raw data supporting the conclusions of this article will be made available by the authors, without undue reservation.

## Ethics statement

The animal study was reviewed and approved by the Institutional Animal Care and Use Committee, New York State Institute for Basic Research in Developmental Disabilities, Staten Island, NY, United States, and the Animal Care Committee, Virginia Polytechnic Institute, Blacksburg, VA, United States.

## Author contributions

JAT conceived and designed the study, obtained the blood and serum specimens, oversaw the execution of the study, and wrote and revised the first draft of the manuscript. MF analyzed the data and wrote the results section. Both authors approved the final manuscript.
